# Human muscle spindles are wired to function as controllable signal-processing devices

**DOI:** 10.7554/eLife.78091

**Published:** 2022-07-13

**Authors:** Michael Dimitriou

**Affiliations:** 1 Physiology Section, Department of Integrative Medical Biology, Umeå University Umeå Sweden; Northwestern University United States; University of Cambridge United Kingdom

**Keywords:** muscle spindle, sensorimotor, proprioception, fusimotor, signal processing, human

## Abstract

Muscle spindles are encapsulated sensory organs found in most of our muscles. Prevalent models of sensorimotor control assume the role of spindles is to reliably encode limb posture and movement. Here, I argue that the traditional view of spindles is outdated. Spindle organs can be tuned by spinal γ motor neurons that receive top-down and peripheral input, including from cutaneous afferents. A new model is presented, viewing γ motor activity as an intermediate coordinate transformation that allows multimodal information to converge on spindles, creating flexible coordinate representations at the level of the peripheral nervous system. That is, I propose that spindles play a unique overarching role in the nervous system: that of a peripheral signal-processing device that flexibly facilitates sensorimotor performance, according to task characteristics. This role is compatible with previous findings and supported by recent studies with naturalistically active humans. Such studies have so far shown that spindle tuning enables the independent preparatory control of reflex muscle stiffness, the selective extraction of information during implicit motor adaptation, and for segmental stretch reflexes to operate in joint space. Incorporation of advanced signal-processing at the periphery may well prove a critical step in the evolution of sensorimotor control theories.

## Introduction

Most of our skeletal muscles contain a large collection of muscle spindle organs. Spindles are generally believed to be basic mechanoreceptors that encode muscle stretch and provide reliable information about actual limb posture and movement kinematics. Previous work and more recent studies using genetic manipulation methods have added a great deal of knowledge about the molecular mechanisms of mechanotransduction (e.g. Kruse and Poppele, 1991; Bewick and Banks, 2015; Woo et al., 2015). Spindles have been proposed to play a basic, low-level role in reflex motor control (Houk, 1976) and proprioception (Goodwin et al., 1972), and their malfunction has been linked to impaired motor coordination (Sainburg et al., 1993). An interesting recent proposition is that the mechanoreceptive part of spindles responds best to force-related variables, as shown in relaxed muscles (Blum et al., 2017). Still, the role of muscle spindle organs in their entirety (i.e. of the mechanoreceptor under in vivo efferent control) has remained unclear(for a recent comprehensive review see Macefield and Knellwolf, 2018).

In the relaxed muscle of the unengaged human, the characteristics of imposed muscle stretch are rather faithfully encoded by the signals of muscle spindle afferents. Specifically, there are two main types of muscle spindle receptors, the primary and the secondary, which give rise to the primary (type Ia) and secondary (type II) afferents, respectively (Boyd and Davidson, 1997). When imposing a ramp-and-hold stretch of the relaxed muscle, type Ia from this muscle are most responsive during muscle stretch, are sensitive to the rate of change of length (i.e., velocity), may encode static length but are silent during muscle shortening. That is, under passive conditions, primaries can be considered to have both a good dynamic and fairly good static muscle-length sensitivity, whereas type II from passive muscle represent good static length sensitivity but a poorer dynamic sensitivity (Edin and Vallbo, 1990a). These response patterns reflect the general view of spindles, which says that type Ia firing encodes static muscle length and the velocity of stretch, and type II encode static muscle length. However, unlike other types of peripheral mechanoreceptors, the spindle organs have their own motor supply in the form of γ motor (‘fusimotor’) neurons (Barker and Chin, 1961; Matthews, 1972). Despite their rich innervation, the overarching role of muscle spindles in sensorimotor control has remained unclear, particularly so in the context of naturalistic active movement.

**Figure 1.**
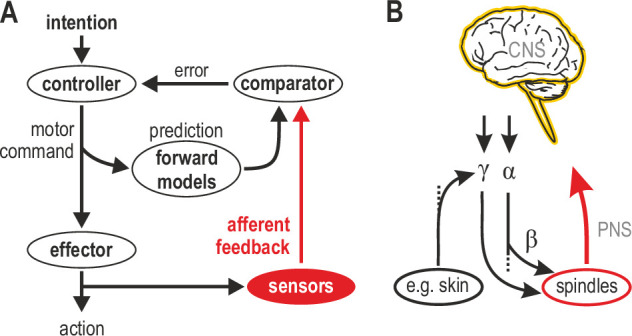
Human sensorimotor control and muscle spindle innervation. (**A**) One prevalent model of human sensorimotor control. Proprioceptors in muscle and skin are viewed as basic sensors, reliably encoding actual mechanical state in unimodal coordinates. Advanced (e.g. selective) processing of sensory signals is thought to occur only in the CNS. (**B**) The role of muscle spindles under naturalistic efferent control has remained unclear. Mammalian muscle spindles can be powerfully controlled by γ motor neurons. These lower motor neurons are subject to both top-down and peripheral control, including from cutaneous afferents. I propose that spindles and their control enable advanced processing of sensorimotor information, giving rise to flexible coordinate representations at the level of the peripheral nervous system (PNS).

Figure 1A represents one prevalent model of how a voluntary movement is controlled and monitored (Wolpert and Miall, 1996). In this model, a controller in the CNS turns the intention to move into a motor command that is sent to skeletal muscles that power the action. A copy of the motor command is sent to internal forward models that make predictions about the sensory consequences of this action. The action itself generates feedback from sensory receptors. If the movement progresses as intended, there should be no discrepancy between the internally predicted signal and actual sensory feedback. This framework views mechanoreceptors in muscle and skin as basic sensors that transduce physical stimuli into unimodal feedback signals, ignoring the independent motor supply to muscle spindles. However, in mammals, ~30% of spinal motor neurons are γ, which supply muscle spindles exclusively (Kuffler et al., 1951; Burke et al., 1977). These γ neurons can be controlled by descending commands and/or peripheral afferent input (Figure 1B; see also following sections). The nervous system has clearly placed a premium on the control of muscle spindle signals at source. Given the renewed emphasis on proprioceptive feedback in motor control (e.g. Crevecoeur et al., 2016; Scott, 2016; Tsay et al., 2021), it is important to strive for a better understanding of how the most complex sensory organ outside of the special senses contributes to sensorimotor function.

A recent debate addressed the independent top-down tuning of human muscle spindles (Burke, 2021a; Burke, 2021b; Dimitriou, 2021a; Dimitriou, 2021b). Here, all possible modes of spindle control are addressed (i.e. independent and α-linked top-down control, as well as peripheral control) to support a unifying proposal: spindles are best thought of as signal-processors that enable flexible coordinate representations at the level of the PNS (Figure 1B). In this framework, spindles can facilitate sensorimotor performance in a flexible manner according to task characteristics, and not limit their contribution to routinely encoding actual posture and movement. In other words, I propose that spindles primarily function for the benefit of sensorimotor performance rather than veridical proprioception. As described in the following sections, this proposal is compatible with previous findings and supported by recent studies where human participants actively engage in fundamental sensorimotor tasks.

### Spindle tuning linked to skeletal muscle activation

The most popularized explanation for human spindle control is based on ‘α-γ co-activation’ (Vallbo, 1970). In this view, γ fusimotor neurons are activated virtually the same time as α motor neurons, in order to prevent spindles from falling slack during muscle contraction. Essentially, in this context, α-γ co-activation simply maintains the stretch sensor operational. That is, the proposed function of fusimotor control is to compensate for the shortcomings/complexities of the neuromuscular system allowing spindles to keep functioning as reliable kinematic proprioceptors. This rather mundane fusimotor function is probably one reason why prevalent computational frameworks have ignored fusimotor control. Most support for a lack of independence between α and γ motor neuron activity has come from recording spindle afferent signals during isometric contractions or during unnaturally slow and restricted movements (e.g. Gandevia and Burke, 1985; Wessberg and Vallbo, 1995; Kakuda et al., 1996). Moreover, co-activation of extrafusal (skeletal) and intrafusal (spindle) muscle fibers can be easily implemented through the more primitive beta neurons (Jami et al., 1982; Emonet-Dénand et al., 1992). β neurons are essentially just α motor neurons that branch out to innervate extrafusal and intrafusal muscle fibers. Both mammals and lower vertebrates have β motor neurons, but only mammals seem to have γ motor neurons (Hunt, 1951; Emonet-Dénand and Laporte, 1975; Murthy, 1978). The vast majority of efferent projections to mammalian spindles are from γ motor neurons (Matthews, 1964; Emonet-Dénand et al., 1992). The independent γ motor supply must therefore represent an evolutionary advantage, realized through the ability to dissociate spindle control from the control of skeletal muscles, in cases where this dissociation is favorable to the organism (see following sections).

**Figure 2.**
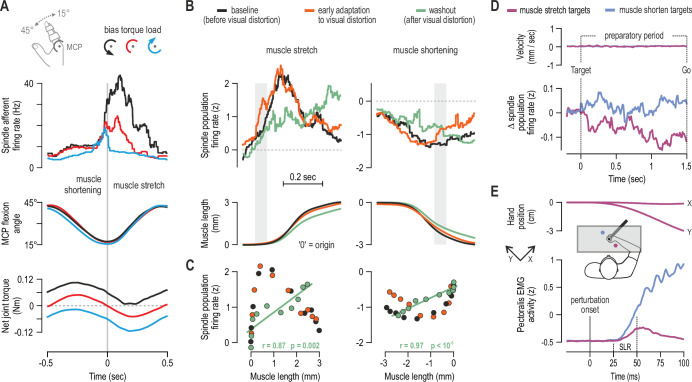
Human muscle spindle organs are not basic kinematic sensors. (**A**) Averaged responses of a representative spindle afferent from the common digit extensor muscle, during active sinusoidal movements of a single finger at 1Hz (adapted from Figure 3 of Dimitriou, 2014). Movement was constrained to the metacarpophalangeal joint (MCP) and occurred under a flexion resistive or assistive torque load, or no external load. Standard classification tests identified the afferent as a typical spindle primary (i.e. ‘type Ia’; see Fig 2 in Dimitriou, 2014). Despite virtually identical finger flexion, spindle responses to stretch varied according to joint dynamics. (**B**) Averaged spindle afferent population responses and equivalent muscle length changes during the classic visuomotor rotation task (both ‘B’ and ‘C’ adapted from Figure 4A and 4E of Dimitriou, 2016). Grey background bars highlight phases in early adaptation (orange) that differ substantially from baseline (black). (**C**) Correlating the signals shown in ‘B’ (down-sampled at 50ms) confirmed a significant relationship in the washout stage. (**D**) Muscle velocity (null) and changes in spindle Ia responses before movement initiation in the classic instructed-delay reaching task with the hand. Ia firing rates from extensor muscles were lower when preparing movement to visual targets associated with stretch of the spindle-bearing muscle (purple). ‘D’ and ‘E’ are adapted from Figure 2B and 6A of Papaioannou and Dimitriou, 2021. (**E**) Averaged signals across participants; experiments using a robotic manipulandum showed a congruent goal-directed tuning of stretch reflexes, including at the short-latency epoch (‘SLR’) in cases where the homonymous muscle was not heavily loaded before perturbation. Color coding as in ‘D’.

Nevertheless, α-γ co-activation can account for the increase in spindle afferent firing observed during isometric contraction of the spindle-bearing muscle (Edin and Vallbo, 1990b); β motor neurons can also contribute (Kakuda et al., 1998). This increase in spindle firing is congruent with the known ‘automatic’ gain-scaling of short-latency stretch reflexes (SLRs), where reflex sensitivity is proportional to background activation of the homonymous muscle, as shown in postural tasks (e.g. Matthews, 1986; Pruszynski et al., 2009). However, automatic gain-scaling alone cannot account for the modulation of SLR gains observed during movement (Dufresne et al., 1980; Soechting et al., 1981; Nakazawa et al., 1997; Wallace and Miles, 1998). I have recently shown that spindle sensitivity to stretch can be positively related to the activity level of the spindle-bearing muscle, but also be negatively related to antagonist muscle activity (Dimitriou, 2014). That is, during continuous sinusoidal movements of a finger against different loads, spindle responsiveness to stretch was shown to depend on the balance of activity across an antagonistic muscle pair (hence joint dynamics), rather than activity in the spindle-bearing muscle alone (Figure 2A). The negative relationship with antagonist activation is compatible with top-down reciprocal inhibition of fusimotor neurons, as shown in intercostal muscles of the cat (Sears, 1964).

Using an innovative experimental approach, Villamar et al., 2021 have very recently tested the hypothesis that SLR sensitivity during movement can be explained by the balance of activity across agonist and antagonist muscles. The observed changes in SLR sensitivity during ballistic elbow movements did reflect the net background activity across agonist and antagonist muscles. Moreover, the relative impact of agonist and antagonist activity on SLR gain were ‘remarkably similar’ to the coefficients generated by the aforementioned spindle study. Although the contribution of other mechanisms cannot be excluded, taken together, the afferent and stretch reflex results suggest that spindle tuning is at least partly responsible for shaping SLR gains during sinusoidal and ballistic movements under different loads. The ‘antagonistic’ mode of control demonstrates that spindle sensitivity to stretch does not only reflect the state of the homonymous muscle. The spindle response to a physical stimulus (i.e. the mechanoreceptor signal) can be modulated or ‘processed’ according to the contractile state of the spindle-bearing muscle and its antagonists. In the context of sinusoidal and ballistic single-joint movement, primary spindles do not seem to function as reliable unimodal sensors encoding muscle stretch or joint rotation (Figure 2A). Rather, by integrating mechanical stimulation and fusimotor commands, spindles help augment volitional motor control according to the prevalent dynamics around a single joint. That is, spindle tuning based on muscle activation balance (i.e. reciprocal control) enables even segmental reflex contribution from single muscles to occur in ‘joint space’. Future research will determine whether spindle tuning can also reflect multi-joint dynamics.

### Independent tuning of muscle spindles in active contexts

As described in the previous section, spindle sensitivity can reflect muscle activation in isometric and movement tasks where differential muscle loading is the predominant or defining variable feature. However, neither ‘α-γ co-activation’ nor ‘antagonistic muscle balance’ can justify the need for an independent fusimotor system. α-linked fusimotor activity could be carried solely by β efferents. So why have we and other mammals evolved γ motor neurons? What is the nature of independent spindle tuning? What are the benefits for sensorimotor performance? With existing methodologies, it has proven virtually impossible to systematically record from human γ motor neurons. Only one study claims to have directly recorded from single γ efferents of immobile humans (Ribot et al., 1986). However, recording from individual spindle afferents using microneurography is a feasible and even preferable alternative, because γ neurons supply spindles exclusively, and the spindle organ acts as an integrator of input from mechanoreception and multiple fusimotor fibers; that is, afferent firing also allows assessment of net fusimotor impact, whereas random fusimotor fibers, whose actions sum non-linearly, may be less revealing in this respect (Matthews, 1972; Prochazka, 1989). Therefore, one way to address the questions above is to record spindle afferent signals during naturalistic movement in fundamental sensorimotor tasks.

One such task involves implicit adaptation to a visual distortion (i.e. the classic visuomotor rotation task). In a recent study, participants used their right hand to perform this task while spindle afferent signals were recorded from wrist extensor muscles (Dimitriou, 2016). The observed adaptation behavior was stereotypical for this type of task: an exponential curve could be fitted to movement direction error in the early adaptation stage and in ‘washout’ (the stage where participants gradually re-adapt to removal of the visual distortion). Despite fundamentally identical muscle kinematics across all stages of the task, there was a dissociation in spindle population signals as a function of task stage. Specifically, compared to baseline, there was an increase in primary muscle spindle sensitivity to stretch in early adaptation (Figure 2B) suggesting a similar increase in stretch reflex gains as a means of reducing movement error online. In the washout stage, spindle afferents (Ia and II) stopped encoding stretch velocity and were instead ‘linearized’ with respect to muscle length (Figure 2BC, green). That is, spindle signals were tuned to hand position only during washout, possibly for facilitating the relevant update of internal models in this stage, where haptic and visual coordinate frames re-align.

In the visuomotor adaptation task, muscle spindles were flexibly tuned according to the need to adapt and the congruence between haptic and visual coordinate frames. A follow-up study applied whole-arm perturbations during probe trials that were randomly interleaved at the different stages of the implicit adaptation task; the study produced equivalent findings concerning stretch reflex tuning, including evidence that levels of SLR attenuation in washout (a proxy for spindle ‘linearization’) reflect individual rates of implicit adaptation (Dimitriou, 2018). It is believed that flexible and adaptive motor control can rely on statistically optimal integration of multimodal sensory inputs (e.g. Körding and Wolpert, 2004; Bays and Wolpert, 2007). For reaching movements, proprioceptive and visual information are thought to be weighted according to their direction-dependent precision (van Beers et al., 1999). Another line of research suggests that the brain constructs flexible coordinate representations depending on task needs and characteristics (Bernier and Grafton, 2010; McGuire and Sabes, 2011; Leoné et al., 2015), although the required coordinate transformations are considered costly due error and noise in the underlying computations (Soechting and Flanders, 1989; Sober and Sabes, 2005; Schlicht and Schrater, 2007). By siphoning multimodal information to the periphery in order to construct flexible representations at source, spindles and their fusimotor control may help alleviate some of the cost associated with internal coordinate transformations.

Another well-studied experimental paradigm is the instructed-delay reach, where there is a delay between a target cue and a ‘Go’ signal to move. This delay is designed to investigate movement preparation. Having a long-enough preparatory delay improves the overall quality of movement and cuts down on reaction time (Rosenbaum, 1980; Ghez et al., 1997; Sutter et al., 2021). Preparatory cortical activity correlates well with parameters such as movement direction/extent and visual target location (Tanji and Evarts, 1976; Weinrich et al., 1984; Kurata, 1993; Shen and Alexander, 1997). It was initially suggested that preparatory cortical activity represents a subthreshold version of the activity seen during movement, but more recent work suggests that preparation sets an initial neural state that somehow facilitates the subsequent movement (Churchland et al., 2010). In a recent study (Papaioannou and Dimitriou, 2021), we demonstrate goal-directed tuning of muscle spindles and stretch reflex gains during movement preparation. Specifically, despite no differences in kinematics or surface EMG during preparation, type Ia firing rates were lower when preparing to reach targets associated with stretch of the spindle-bearing muscle (Figure 2D). That is, spindle responses can also be flexibly adjusted according to ‘extrinsic’ visual information about target location. These findings are congruent with recent reports of preparatory modulation in the primary somatosensory cortex (Ariani et al., 2022; Gale et al., 2021), but suggest that such preparatory changes in the CNS may be partially due to processing altered afferent signals, rather than exclusively reflect internally-generated commands or priming.

We also found a strong positive relationship between type Ia firing during late preparation and time-to-peak velocity during reaching, suggesting that spindle preparatory tuning has a substantial impact on the subsequent voluntary movement (Papaioannou and Dimitriou, 2021); every additional unit increase in Ia firing rate involved a 3ms delay in attaining peak velocity during movement. This relationship can be understood in terms of the spindle’s role in stretch reflexes. By independently modifying spindle gains, the fusimotor system can affect the degree of reflex muscle stiffness during movement execution, without affecting contractile muscle force during preparation. Modulating the level of reflex stiffness in a goal-appropriate manner can facilitate the execution of planned reaching movements. Muscle afferent (reflex) feedback contributes significantly to force generation, about a third of volitional contraction (Hagbarth et al., 1986; Gandevia et al., 1990), regardless if the contraction is maximal or not (Macefield et al., 1993).

It is known that spindle Ia signals can also affect long-latency stretch reflex responses - LLRs (e.g. Hunter et al., 1988; Fellows et al., 1993; Pruszynski and Scott, 2012). Additional experiments implicating whole-arm perturbations confirmed that goal-directed tuning of type Ia responses reflected a congruent modulation of stretch reflex gains at all latencies, including at SLR latencies in cases where the muscle was not heavily pre-loaded (Figure 2E). LLR gains exhibited goal-dependency regardless of muscle pre-loading level (Papaioannou and Dimitriou, 2021). The same study demonstrated that goal-directed modulation of LLR gains was stronger following a long rather than a relatively short preparatory delay, closely matching the temporal evolution of spindle preparatory tuning. Moreover, the used ‘short’ preparatory delay (200–250 ms) is considerably longer than the minimum delay required for shaping LLR responses via selective CNS processing (e.g. Yang et al., 2011; Scott, 2016), but shorter than the time required for full afferent expression of changes in dynamic fusimotor drive (Crowe and Matthews, 1964). Future work will determine whether spindle tuning helps control reflex muscle stiffness across different tasks (such as object interception), and further clarify how muscle loading relates to possible independent tuning of spindles. For example, one approach could involve examining spindle afferent responses during dynamic (‘force-field’) learning (Shadmehr and Mussa-Ivaldi, 1994).

Nevertheless, in planned voluntary reach, spindle responses to stretch can be locally adjusted (or ‘processed’) according to the intention to move in a particular direction (Figure 2D). That is, tuning of human spindles can reflect specific goals within a behavioral context (reaching), which represents a finer degree of spindle modulation than tuning according to behavioral context or type of task, as previously and more recently suggested (Prochazka et al., 1985; Ribot-Ciscar and Ackerley, 2021). One study found no evidence of a selective effect on fusimotor neurons when anticipating the need to make a contraction that would oppose an imposed movement of the foot at the ankle (Burke et al., 1980). However, our 2021 study was the first to implicate true reaching intention and action. In this case, the intention to perform a voluntary goal-directed movement may be necessary for engaging independent fusimotor control.

It should be emphasized that all findings described in Figure 2 involve control of the dominant upper limb. It is possible that there is a large degree of functional specialization in the fusimotor control of upper vs. lower limbs. Most of what we know concerning mammalian muscle spindle structure and fusimotor function has come from work with cats (e.g. Barker, 1948; Matthews, 1972; Hulliger, 1984), and many inferences we currently make concerning human fusimotor control would have been impossible without this work. For example, such research has shown that there are two independently controlled groups of γ motor neurons, ‘static’ and ‘dynamic’, with the latter innervating only primary muscle spindles (Matthews, 1962). In active cats, fusimotor and spindle activity has been most thoroughly examined in the context of locomotion (see e.g. Prochazka, 1996). Equivalent data during human locomotion are lacking due to methodological limitations. Mathematical modelling suggests that fusimotor control optimizes the spindles’ ability to encode position sense by accounting for the presence of musculoskeletal complexities and output noise (Scott and Loeb, 1994). However, another prominent line of work suggests that spindles are not length detectors, but instead are independently controlled in a predictive manner in order to modulate the function of spinal central pattern generator (CPG) circuits during locomotion (Ellaway et al., 2015). A similar fusimotor support of human bipedal locomotion may occur, although it is currently unclear whether CPG networks exist in the human spinal cord (Minassian et al., 2017). Recordings from humans have also suggested a more predictive role for spindle signals. In one paper, we correlated spindle population responses recorded during block-grasping and key-pressing with muscle velocity occurring at the same time as the recorded afferent signal, and velocity observed at different points into the future (Dimitriou and Edin, 2010). The closest relationship was between afferent firing rate and velocity ~150ms after the spindle signal. This result meant that muscle spindles fulfilled all three neurophysiological criteria for identifying a forward sensory model (Wolpert and Miall, 1996): spindle inputs were the current state of the system (mechanoreception) and an efferent command (β or α-γ), and spindle output predicted the future kinematic state. However, later studies showed that the spindles’ ‘predictive’ capacity does not hold across tasks. For example, if anything, the opposite results should have been observed in preparatory modulation (e.g., Figure 2D), and suddenly adding a new external load did not significantly alter the predictive capacity of spindles during the initial cycles of sinusoidal movement (Dimitriou, 2014). To identify forward sensory models, one could perhaps add a fourth criterion stating that forward models should make worse predictions in novel contexts.

### Peripheral control of spindle sensitivity

Prominent theories of spindle and fusimotor control have not incorporated the possibility of substantial afferent influence on fusimotor neurons. Peripheral (‘reflexive’) input to γ neurons, including from cutaneous afferents, has been mainly demonstrated using electrical nerve stimulation in anaesthetized cats (Appelberg et al., 1977; Johansson and Sojka, 1985; Johansson et al., 1986). These findings reinforce the idea that pools of γ motor neurons should be considered as an integrative system able to combine sensorimotor ‘apples and oranges’, that is, descending commands and peripheral multisensory information.

A functional degree of peripheral multisensory integration – such as for dexterous object manipulation – may be possible at the level of the spindle as a result of afferent control of γ neurons. This hypothesis is compatible with evidence of ‘multimodal’ signals (tactile-proprioceptive) already in area 3a of the somatosensory cortex (Kim et al., 2015). However, so far, cutaneous stimulation has been shown to have a limited impact in two studies of spindle afferent activity in passive humans (Aniss et al., 1990; Gandevia et al., 1994). But afferent control of spindle sensitivity may prove stronger or more easily unmasked in the active individual (e.g. due to higher background tonus). The specific functional advantage of having such afferent connections is currently unclear. One previous study has demonstrated edge-orientation processing in tactile neurons as a function of their receptive fields (Pruszynski and Johansson, 2014). However, in this case, cutaneous afferent signals were bound to the characteristics (edge-orientation) of the ‘adequate’ physical stimulus. In contrast, muscle spindle output can potentially be modulated according to the characteristics of a physical stimulus in another modality (e.g. cutaneous), via efferent control. Although top-down control alone supports the notion that spindles are best viewed as flexible signal-processing devices rather than basic mechanoreceptors (Figure 1B and Figure 2), the possibility of substantial peripheral control of fusimotor neurons adds another layer of support to this proposition.

**Figure 3.**
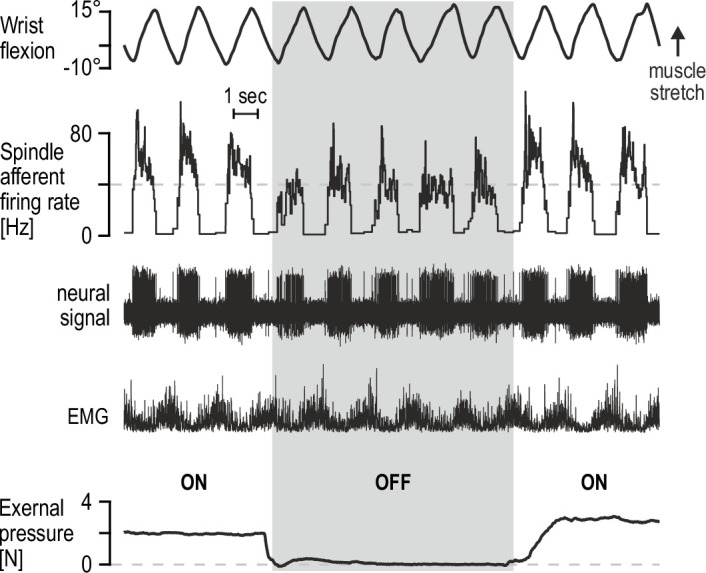
Percutaneous mechanical pressure near the spindle capsule affects encoding of active movement. Responses of a spindle afferent from a wrist extensor muscle while the participant continuously moved their right semipronated hand about the wrist (flexion-extension; 0° denotes alignment with forearm). A hand-held probe was used by the experimenter for applying and measuring mechanical pressure over a small area of skin on the forearm (5 mm probe tip diameter), near the spindle capsule, during some movement cycles only (grey vertical bar denotes stimulus removal). Throughout, the participant’s gaze was directed at a monitor displaying a cursor that tracked hand movement. Despite very similar hand movement and activation patterns of the spindle-bearing muscle (‘EMG’), spindle responses to hand flexion were markedly stronger during percutaneous pressure.

While fusimotor innervation can allow spindles to function as controllable signal-processors, peripheral modulation of spindle sensitivity may be also enabled by the structure of these encapsulated organs. It has long been known in microneurography circles that percutaneous mechanical pressure applied near the spindle capsule, likely leading to its compression, can have some effect on spindle afferent firing. Such mechanical pressure can otherwise represent an ecologically valid stimulus, brought on by increased intramuscular pressure due to active contraction or simply materialize when muscles are palpated. Representative preliminary data from our lab indicate that, regardless of underlying mechanism, spindle afferent responses to active movement are substantially affected by light-to-moderate percutaneous pressure applied near the spindle capsule (Figure 3). It is tempting to speculate as to the potential regulatory function of such peripheral modulation, especially in the context of recent findings that intramuscular fluid pressure can have immediate and significant effects on contractile muscle force (Sleboda and Roberts, 2020). Nevertheless, the preliminary findings in Figure 3 serve as yet another indication that spindles are inclined to produce flexible representations rather than a consistent picture of actual limb kinematics.

### Concluding remarks

**Figure 4.**
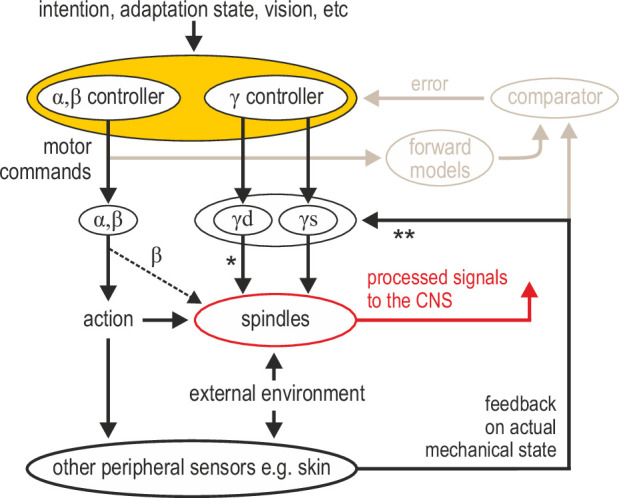
Advanced signal-processing at the level of muscle spindle organs. In addition to descending commands to skeletal muscles and an efferent copy to forward models (Figure 1A), there can be independent descending control of γ dynamic (‘γd’) and γ static (‘γs’) spinal motor neurons. The vast majority of efferent projections to spindles are from γ motor (‘fusimotor’) neurons, but there is also some β supply (indicated by the thinner dashed line). Fusimotor control can affect spindle output in the absence of mechanical stimulation (e.g. muscle stretch), but fusimotor activity can also shape spindle responses to direct mechanical stimulation arising from own action or the external environment. ‘*’: γd project only to primary muscle spindles, allowing for differential control of primary and secondary muscle spindles. Electrophysiological studies in mammals have also demonstrated multisensory afferent convergence onto fusimotor neurons. ‘**’: The specific impact of afferent control of fusimotor neurons has not been determined yet in the active human, and may well vary across body segments e.g., stronger in the hand and/or the foot. In this model, joint and cutaneous receptors (and vision) provide consistent/reliable information about actual bodily state, and potentially so do spindles, e.g., if they are predominantly affected by direct mechanical stimulation (as in the case of the passive, unengaged individual). But here, fusimotor activity represents an intermediate coordinate transformation that allows multimodal information to converge on spindles, creating flexible representations at the periphery. So far, spindle tuning has been shown to facilitate load compensation in joint space, the selective extraction of information during motor adaptation, and the independent preparatory adjustment of reflexive muscle stiffness before goal-directed reaching (Figure 2).

I propose that muscle spindle organs are versatile signal-processing devices whose overarching role is to facilitate sensorimotor performance according to task characteristics, rather than faithfully encode posture and movement. Here, I have outlined recent evidence that spindle tuning can enable the independent preparatory control of muscle compliance, the selective extraction of information during implicit motor adaptation, and for segmental stretch reflexes to operate in joint space. The complete spindle repertoire remains to be revealed. Of particular interest is the ability of spindles to act as conduits of multimodal information. The fusimotor neurons controlling spindles can integrate multisensory peripheral input and top-down commands (which can also reflect sensory events, e.g. in vision; Figure 2D). It is reasonable to think of fusimotor activity as an intermediate coordinate transformation enabling different information to converge on spindles, generating flexible coordinate representations at level of the PNS (Figure 4). Such dimensionality reduction may potentially simplify motor control without limiting performance. A more flexible and central role for spindles justifies the premium placed on their control by the nervous system (i.e. ~30% of lower motor neurons are γ). Such a role is also compatible with the seemingly large number of parameters found to correlate closely with motor and premotor neural activity, and with models that claim the motor cortex essentially operates in ‘proprioceptive’ coordinates (Adams et al., 2013).

Consistent information about actual limb position and movement kinematics is also necessary. It is widely believed that how we sense our body, including its position and movement, depends on the interplay of multimodal signals (e.g. Makin et al., 2008; Proske and Gandevia, 2012). While spindles can encode limb position preferentially in certain contexts (e.g. Figure 2C), vision, joint and cutaneous signals also contribute to proprioception and kinesthesia (Collins and Prochazka, 1996; Collins et al., 2005; Sarlegna and Sainburg, 2009). Both a flexible role for spindles and multimodal contributions to proprioception are supported by the general model proposed here (Figure 4). Interestingly, it is known that direct electrical stimulation of single joint and cutaneous afferents evokes appropriate sensations, but stimulation of single spindle afferents does not lead to any conscious sensations in the absence of movement (Macefield et al., 1990). Tendon vibration (artificial spindle stimulus) of the unseen limb can lead to illusory perception of physically impossible limb configurations, and seeing the vibrated limb strongly attenuates illusory motion (Lackner and Taublieb, 1984). If spindles are not routinely tasked with providing a faithful representation of posture and limb kinematics (i.e., not tasked with encoding the consequences of action), spindle tuning can instead emphasize the flexible facilitation of concurrent or future action. While recording from human afferents and performing follow-up behavioral studies has helped shape our understanding of spindle function, elucidating the underlying mechanisms in more detail will require much more work on multiple fronts. For example, predictions stemming from human afferent data concerning fusimotor function can be tested more freely in animal models, using a range of modern techniques, as recently emphasized (Wilkinson, 2021). Achieving a comprehensive account of spindle contribution will likely also advance our understanding of core sensorimotor principles.

### Open questions

1. Does the nervous system tune muscle spindles according to multi-joint dynamics?
2. Beyond planned reaching, does independent tuning of spindles help control muscle compliance across different tasks (e.g., object interception)?
3. In terms of task-relevant flexibility, how different is the tuning of primary and secondary muscle spindle receptors?
4. Given the peripheral afferent input to fusimotor neurons, does cutaneous stimulation have a significant impact on spindle sensitivity in the active human? What are the benefits for sensorimotor performance e.g., in terms of the dexterous manipulation of objects?
5. Is there substantial functional specialization in spindle tuning across human upper and lower limbs (e.g. in the degree of cutaneous modulation), and if so, what is its purpose? Similarly, are there differences in spindle control between the dominant and non-dominant limb, and can such differences account for discrepancies in sensorimotor performance?
6. Which brain areas and descending pathways are involved in fusimotor control during e.g., movement preparation?
